# Patient and operative factors associated with unanticipated intensive care admission and outcomes following posterior fossa decompressions in children: A retrospective study

**DOI:** 10.1111/pan.14496

**Published:** 2022-06-03

**Authors:** Hubert A. Benzon, Anthony Tantoco, Anthony Longhini, John Hajduk, Amanda Saratsis, Santhanam Suresh, Robert J. McCarthy, Narasimhan Jagannathan

**Affiliations:** ^1^ Department of Pediatric Anesthesiology, Ann & Robert H. Lurie Children's Hospital of Chicago Northwestern University Feinberg School of Medicine Chicago Illinois USA; ^2^ Department of Anesthesiology Yale University School of Medicine New Haven Connecticut USA; ^3^ Department of Neurological Surgery Riley Hospital for Children, and Indiana University School of Medicine Indianapolis Indiana USA; ^4^ Department of Anesthesiology Rush University Chicago Illinois USA

**Keywords:** child, complications, general anesthesia, neurosurgery

## Abstract

**Introduction:**

Posterior fossa decompression for Chiari I Malformation is a common pediatric neurosurgical procedure. We sought to identify the impact of anesthesia‐related intraoperative complications on unanticipated admission to the intensive care unit and outcomes following posterior fossa decompression.

**Methods:**

Medical records of all patients <18 years who underwent surgery for Chiari I malformation between 1/1/09 and 1/31/21 at the Ann & Robert H. Lurie Children's Hospital of Chicago were included. Records were reviewed for patient characteristics, anesthesia‐related intraoperative complications, postoperative complications, and surgical outcomes. The primary outcome was the incidence of unanticipated admission to the intensive care unit, and the primary variable of interest was an anesthesia‐related intraoperative complication. Patient, surgical characteristics, and year of surgery were also compared between patients with and without an unanticipated admission to the intensive care unit, and a multi‐variable adjusted estimate of odds of unanticipated admission to the intensive care unit admission following an anesthesia‐related intraoperative complication was performed. Secondary outcomes included anesthesia factors associated with an anesthesia‐related intraoperative event, and postoperative complications and surgical outcomes between patients admitted to the intensive care unit and those who were not.

**Results:**

Two hundred ninety‐six patients with Chiari I Malformation were identified. Clinical characteristics associated with an unanticipated admission to the intensive care unit were younger age, American Society of Anesthesiologist (ASA) physical status >2 and an anesthesia‐related intraoperative complication. 29 anesthesia‐related intraoperative complications were observed in 25 patients (8.4%). Two of 25 patients (8%) with an anesthesia‐related intraoperative complication compared with 3 of 271 (1%) patients without anesthesia‐related intraoperative complication had an unanticipated admission to the intensive care unit, odds ratio 7.8 (95% CI 1.2–48.8, *p* = .010). When adjusted for age, sex, ASA physical status, presenting symptoms, concomitant syringomyelia, previous decompression surgery and year of surgery, the odds ratio for an unanticipated admission to the intensive care unit following an anesthesia‐related intraoperative complication was 5.9 (95% CI 0.51–59.6, *p* = .149). There were no differences in surgical outcomes between patients with or without an unanticipated admission to the intensive care unit.

**Conclusion:**

Our study demonstrates that although anesthesia‐related intraoperative complications during posterior fossa decompression are infrequent, they are associated with an increased risk of an unanticipated admission to the intensive care unit.

What is already known about the topic

Posterior fossa decompression for Chiari I Malformation is a common neurosurgical procedure in pediatric anesthesia practice, but the impact of anesthesia‐related intraoperative complications on the incidence of unanticipated intensive care admission and 30‐day outcomes is unknown.

What new information this study adds

This study confirmed the rate of anesthesia‐related complications during posterior fossa decompression in children was low (<10%); however, 2 of 25 patients with an anesthesia‐related intraoperative complication (8%) compared with 3 of 271 (1%) without anesthesia‐related intraoperative complication had an unanticipated admission to the intensive care unit, unadjusted odds ratio 7.8 (95% CI 1.2–48.8, *p* = .010) and an adjusted odds ratio of 5.9 (0.51–59.6, *p* = .149). Thirty‐day outcomes were not different in patients with or without an unanticipated admission to the intensive care unit.

## 1 INTRODUCTION

Studies reporting the effects of patient and surgical factors on perioperative complication rates for children undergoing neurosurgical operation including craniosynostosis repair,^1^, ^2^, ^3^ epilepsy surgery,^4^ and other neurosurgical procedures (for hydrocephalus, spinal and cranial anomalies, brain tumor, cerebellar cancer, and trauma)^5^ appear in the literature. A 2015 review of publications on posterior fossa decompression for Chiari I Malformation, a common pediatric neurosurgical procedure, revealed 145 articles, with 39 specific to the pediatric population, 22 of which reported patient complications.^6^ Importantly, all 22 of these studies were published in neurosurgery journals, with an emphasis on surgical complications and outcomes. Indeed, there are no studies that primarily evaluate anesthetic‐related complications and outcomes in children who undergo posterior fossa decompression for Chiari I Malformation.

For this reason, we performed a retrospective study to examine clinical characteristics and intraoperative and postoperative adverse events for this surgical patient population. Our overall aim was to identify patient and operative predictors and specifically the impact of an anesthesia‐related intraoperative complication on the odds of an unanticipated admission to the intensive care unit as a marker of a clinically significant adverse event in this population. Knowledge of these factors may allow for improved anesthetic planning and management in order to reduce and prepare for these complications. Specifically, we hypothesized that intraoperative anesthesia‐related complications would occur in approximately 10% of patients,^5^ and that an intraoperative anesthesia‐related complication would increase the odds ratio for an unanticipated intensive care unit admission following posterior fossa decompression for Chiari I Malformation.

## 2 MATERIALS AND METHODS

Approval for this retrospective chart review study was granted by the Institutional Review Board of the Ann & Robert H. Lurie Children's Hospital of Chicago (IRB 2016–110). A waiver of informed consent was granted by the IRB as the study was deemed of minimal risk to subjects, and data were acquired from preexisting records. The hospital records of all patients aged less than 18 years old who underwent a posterior fossa decompression for Chiari I Malformation over a 12‐year span between 1/1/2009 and 1/31/2021 at the Ann & Robert H. Lurie Children's Hospital of Chicago were reviewed. The start date of 1/1/2009 was chosen as this was the initiation date of electronic medical records at our institution. Patients were excluded from data collection and analysis if they were designated as an emergency procedure or combined with another surgical procedure.

Patient medical, anesthesia, and surgical records were queried for the following: demographic and clinical variables including age, weight, height, gender, surgical diagnosis, initial presenting symptoms leading to diagnosis of Chiari I malformation, and medical comorbidities (cardiac, pulmonary, renal, neurologic); evidence of upper respiratory infection; prior perioperative anesthetic history (nausea/vomiting, difficult airway); American Society of Anesthesiologists (ASA) physical status classification system; and preoperative clinical and laboratory test results. Intraoperative variables were abstracted from the anesthesia records, including but not limited to induction type, medications used, fluid administration, estimated blood loss, changes in neuromonitoring signals, duration of procedure, and any complication or adverse clinical event. Intraoperative anesthesia‐related complications were defined before data collection and considered relevant when an intervention was needed beyond titration of anesthetic agents. These included any cardiac event requiring medication intervention (bradycardia, hypotension, hypertension, or arrhythmias requiring medication treatment such as atropine, phenylephrine, labetalol, or lidocaine, respectively), desaturations defined as a peripheral oxygen saturation less than 93% for greater than 10 min, blood product transfusion, naloxone administration for sedation, airway adjustment while in prone position (readjustment of endotracheal tube placement, reintubation, etc.) needed, or possible venous air embolism.

Postoperative data extracted from the medical records included admission to the intensive care unit (either pediatric or neonatal intensive care unit) and postoperative complications that occurred during hospital stay including a cardiac event requiring medication administration (bradycardia, hypotension, hypertension, or arrhythmias requiring medication treatment as mentioned previously), respiratory (unexpected postoperative intubation, apnea requiring oxygen intervention, significant airway obstruction requiring intervention, aspiration, or pneumonia), blood product transfusion, infection requiring antibiotic administration, documented nausea/vomiting requiring medication administration, and uncontrolled pain defined as a pain score equal to or greater than 7 on an age‐appropriate validated pain scale.

Surgical outcomes were assessed from the time of procedure completion to the point of longest clinical follow‐up after hospital discharge with the operating surgeon available, in the medical record. The following factors were assessed: neurological improvement; new or worsening neurological deficit; resolution of a syrinx; development of a pseudomeningocele; aseptic meningitis; cerebrospinal fluid leak; and wound infection. Patient charts were reviewed for emergency room visits within 7 days of surgery and readmission within 30 days of discharge.

### 2.1 Statistical analysis

The primary outcome assessed was the incidence of an unanticipated admission to the intensive care unit. The primary variable of interest was an anesthesia‐related intraoperative complication. Patient characteristics (age, sex, body mass index [BMI], ASA physical status, cervical spine instability, a syndrome diagnosis), presenting symptoms (incidental, neurological, comorbidity), surgical factors (history of prior posterior fossa decompression surgery, concomitant syringomyelia, size of the malformation, and duraplasty), year of surgery, and intraoperative anesthetic‐related complications were compared between patients with and without an unanticipated admission to the intensive care unit. Unadjusted comparisons between groups for interval data were made using the Mann–Whitney U‐test, with ordinal and nominal data compared using a continuity corrected chi‐squared analysis or Fisher's exact test. The Cochran–Armitage test for trends was also performed to assess trends in the incidence of an unanticipated admission to the intensive care unit as well as an intraoperative anesthesia‐related complication across the years of the study. A multi‐variable adjusted estimate of the odds of an unanticipated admission to the intensive care unit admission following an intraoperative anesthetic complication was made using a logistic regression model using Firth's bias reduction method. Variables that demonstrated an effect size >0.1 on univariable analysis were entered into the model. Odds ratios are expressed as the exponent of beta with 95% confidence intervals of the estimates determined by penalized profile likelihood method (R package logistf). The unadjusted and confounder adjusted number needed to harm was calculated as 1/(risk of an unanticipated intensive care unit admission times the risk of an anesthesia intraoperative event).

Secondary outcomes included intraoperative anesthesia variables associated with an anesthesia‐related intraoperative event, and postoperative complications and surgical outcomes between patients admitted to the intensive care unit and those who were not were compared using a chi‐squared statistic or the Mann–Whitney U‐test. Differences and confidence intervals for the difference in the rate of postoperative complications and surgical outcomes among groups were calculated using the Coppler–Pearson method for binary outcomes. Median differences and 95% confidence intervals for continuous data were calculated using a 10 000‐sample bootstrap. The sample for this study was all patients undergoing a posterior fossa decompression surgery between 1/1/2009 and 1/31/2021. Assuming 24 subjects per year for 12 years, a minimum of 288 patients would be included in the study a logistic regression of the unanticipated admission to the intensive care unit as a primary response variable on the binary predictor variable of which 10% of subjects would exhibit an intraoperative anesthesia event, would achieve 90% power to detect at the 0.05 significance level the change in probability of an intensive care unit admission from 0.02 to 0.075. This difference corresponds to an odds ratio of 4.0. A two‐sided Wald test was used for this estimate.

Statistical analysis was performed using RStudio version 2021.09.0 Build 351 (Integrated Development for R. RStudio, Inc.; URL: http://www.rstudio.com/) and R version 4.1.1, release date 8/10/2021 (The R Foundation for Statistical Computing). Sample size analysis was performed using PASS 15.0.13, release date 2/10/2020 (NCSS, LLC, Kaysville UT).

## 3 RESULTS

Two hundred ninety‐six patients who underwent posterior decompression surgery for Chiari I Malformation were identified. One hundred and eighty‐one children were female (61%), with a median (IQR) age of 10^5^, ^6^, ^7^, ^8^, ^9^, ^10^, ^11^, ^12^, ^13^, ^14^ years. The primary factors leading to a Chiari I diagnosis were neurological symptoms (182 of 296, 62%), followed by symptoms of a clinical comorbidity (92 of 296, 31%), and an incidental radiological finding (22 of 296, 7%) (Table S1 in Appendix S1). Duraplasty was performed in 192 of the 296 cases (65%).

Five patients (5 of 296, 1.7%) had an unanticipated intensive care unit admission following surgery, and thirteen patients had a planned admission to the intensive care unit. Univariable analysis of the clinical characteristics associated with an unanticipated admission to the intensive care unit are shown in Table 1. There was no difference in the frequency of an unanticipated intensive care unit admission (chi‐squared 6.698, *p* = .877 or Cochran–Armitage test for trend z = 1.507, *p* = .122) across the years of the study. Younger age, ASA physical status >2 and an intraoperative anesthesia‐related complication were greater in patients with an unanticipated admission to the intensive care unit on univariable analysis.

**TABLE 1:** Clinical characteristics, intraoperative anesthesia complications, surgical factor, and year of surgery in subjects with and without an unanticipated intensive care unit admission following posterior fossa decompression surgery for Chiari I Malformation

	Unanticipated intensive care unit admission		*p*	Effect size (95% Confidence interval)
	No (*n* = 291)	Yes (*n* = 5)		
Age (y)	10 (5–14)	3 (1–7.5)	.016	1.08 (0.19–1.97)
BMI (kg/m^2^)	18.7 (15.5–22.3)	18.6 (15.4–29.6)	.972	−0.14 (−1.03–0.73)
Sex				
Female	177 (61)	4 (80)	.652	0.19 (−0.20 to 0.53)
Male	114 (39)	1 (20)		
ASA physical status				
1 or 2	226 (78)	0 (0)	<.001	−0.77 (−0.82 to −0.72)
3 or 4	65 (22)	5 (100)		
Presenting symptom				
Incidental	21 (7)	1 (20)	.144	−0.18 (−0.67 to 0.42)
Neurological	181 (62)	1 (20)		
Comorbidity	89 (31)	3 (60)		
Cervical spine instability	5 (2)	0 (0)	.804	0.02 (0.00–0.03)
Syndrome^a^	17 (6)	0 (0)	.613	0.06 (0.03–0.08)
Previous decompression surgery	24 (8)	1 (20)	.356	−0.11 (−0.46 to 0.26)
Size of malformation (mm)^b^	10 (7–15)	11.5 (6–26)	.684	−0.59 (−1.58 to 0.40)
Concomitant syringomyelia	168 (57)	1 (20)	.168	0.38 (−0.05 to 0.69)
Dura entered	189 (65)	3 (60)	.818	0.04 (−0.39 to 0.47)
Duration of surgery (min)	189 (149–225)	129 (98–308)	.719	−0.01 (−0.89 to 0.88)
Intraoperative complication				
No	268 (92)	3 (60)	.010	−0.32 (−0.69 to 0.18)
Yes	23 (8)	2 (40)		
Intraoperative complication count by type				
Bradycardia	3	0	.819	0.01 (−0.00 to 0.02)
Hypertension	2	0	.852	0.00 (−0.0 to 0.01)
Hypotension	3	1	.066	−0.19 (−0.52 to 0.20)
Arrythmia	0	0	‐‐	‐‐
Aspiration	0	0	‐‐	‐‐
Transfusion	2	0	.852	0.01 (−0.00 to 0.02)
Hypoxemia	0	0	‐‐	‐‐
Oversedation	6	0	.746	0.02 (0.00–0.03)
Loss of Neuromonitoring Signals	3	1	.066	−0.19 (−0.52 to 0.20)
Airway Adjustment	6	0	.746	0.02 (0.00–0.04)
Unanticipated Extubation	1	0	.896	0.00 (−0.00 to 0.01)
Possible Venous Air Embolism	0	1	.017	−0.2 (−0.38 to −0.01)
Year of surgery				
2009–2010	42 (14)	1 (20)	.877	0.41 (0.04–0.68)
2011–2012	43 (15)	1 (20)		
2013–2014	49 (17)	2 (40)		
2015–2016	61 (21)	1 (20)		
2017–2018	59 (20)	0 (0)		
2019–2021	37 (13)	0 (0)		

*Note*: Data presented as median (1st–3rd quartile) or *n* (% of column). Effect size reported as Hedges g for continuous data or Cliff's Delta for nominal or ordinal data.

Abbreviations: ASA, American society of anesthesiologists; BMI, body mass index.

^a^ Syndromes included neurofibromatosis 1, Xia‐Gibbs syndrome, Goldenhar, DiGeorge, Mucopolysaccharidosis type 4 (Morquio), Pfeiffer Syndrome.

^b^ Number with reported malformation size (*n* = 204).

Twenty‐nine anesthesia‐related intraoperative complications were observed in 25 patients (8.4%). There was no difference in the frequency of an anesthesia‐related intraoperative complications (chi‐squared 14.222, *p* = .287 or Cochran–Armitage test for trend z = −0.897, *p* = .369) across the years of the study. Intraoperative cardiovascular complications included bradycardia (*n* = 3), hypotension (*n* = 4), and hypertension (*n* = 2). One potential venous air embolism was diagnosed clinically due to sudden drop in end‐tidal CO_2_ with hemodynamic changes, which resolved without sequelae. Six patients while in the prone position required adjustments in their airway (three for repositioning of endotracheal tube due to mainstem intubation, two patients requiring flip back to supine position for reintubation due to obstruction, and one kinked tube that resolved after evaluation with fiber‐optic), and six patients were given naloxone for over‐sedation noted during emergence while trying to extubate. Two patients received intraoperative red blood cell transfusion. Lastly, four of 142 patients with neuromonitoring had decreased SSEP signals: two resolved spontaneously, one improved after nitrous oxide was turned off, and one improved after a propofol drip was initiated and the inhalational agent was discontinued. Intraoperative anesthesia‐related complications were not greater in patients who had a duraplasty (19 of 192, 9.9%) compared with patients who did not (6 of 104, 5.8%), difference 4.1%, 95% CI −2.8% to 11.0%, *p* = .277.

All patients received general anesthesia with inhalation maintenance, and 95% received intraoperative neuromuscular blockade. A total of 291 patients (98%) received intraoperative opioids, with 47 (16%) receiving intraoperative dexmedetomidine. The association of anesthesia factors with an intraoperative anesthesia‐related event is shown in Table 2.

**TABLE 2:** Anesthesia factors associated with an intraoperative anesthesia‐related complication during posterior fossa decompression surgery for Chiari I Malformation

	Intraoperative anesthetic‐related complication		*p*	Effect size (95% Confidence interval)
	No (*n* = 271)	Yes (*n* = 25)		
Induction type				
Inhalational	185 (68)	16 (64)	.659	0.09 (−0.32 to 0.50)
Intravenous	86 (32)	9 (36)		
Maintenance inhalational agent				
Sevoflurane	250 (92)	20 (80)	.027	−0.11 (−0.27 to 0.04)
Isoflurane	16 (6)	5 (20)		
Sevoflurane / Isoflurane combined	5 (2)	0 (0)		
Nitrous Oxide	9 (3)	4 (16)	.017	−0.13 (−0.27 to 0.02)
Local anesthetic				
None	131 (48)	8 (32)	.219	−0.19 (−0.39 to 0.03)
Per anesthesia	53 (20)	5 (32)		
Per Surgery	87 (32)	12 (48)		
Neuromuscular blockade	256 (94)	25 (100)	.625	−0.05 (−0.08 to −0.03)
Opioids				
Any	266 (98)	25 (100)	.493	0.13 (−0.04 to 0.29)
Fentanyl bolus	198 (73)	22 (88)	.149	−0.14 (−0.28 to 0.00)
Fentanyl infusion	65 (24)	6 (24)	.999	−0.00 (−0.17 to 0.17)
Remifentanil infusion	38 (14)	2 (8)	.549	0.06 (−0.05 to 0.17)
Dexmedetomidine infusion	42 (16)	5 (20)	.581	−0.04 (−0.02 to 0.12)
Propofol infusion	44 (16)	3 (12)	.327	0.04 (−0.09 to 0.17)
Intraoperative fluids (mls/kg/hr)	10 (7–14)	11 (8–14)	.340	−0.08 (−0.48 to 0.33)
Estimated blood loss (ml)^a^	25 (10–50)	20 (10–50)	.233	0.20 (−0.20 to 0.61)
Arterial line	87 (32)	11 (44)	.268	−0.11 (−0.31 to 0.09)

*Note*: Data presented as median (1st–3rd quartile) or *n* (% of column). Effect size reported as Hedges g for continuous data or Cliff's Delta for nominal or ordinal data.

^a^ Number with recorded estimate blood loss (*n* = 274).

Two of the 25 (8.0%) patients with an intraoperative anesthesia‐related complication compared with 3 of 271 (1.1%) patients without anesthesia‐related intraoperative complication had an unanticipated admission to the intensive care unit, odds ratio 7.8 (95% CI 1.2–48.9, *p* = .010). Intraoperative anesthesia‐related complication in the 2 patients with an unanticipated admission to the intensive care unit included a patient with a history of mitral valve regurgitation and tricuspid valve regurgitation and failure to thrive who had hypotension requiring a vasopressor with a potential intraoperative venous air embolism, and a patient with a history of transposition of the great arteries after cardiac surgery and chronic kidney disease with vesico ureteral reflux and aspiration pneumonia who had decreased SSEP signals intraoperatively. Three patients without an intraoperative anesthesia complication had unanticipated intensive care unit admissions. One patient had a history of obstructive sleep apnea requiring continuous positive airway pressure (CPAP) after surgery, and the other two patients had known vocal cord paralysis and laryngomalacia with recent respiratory infections with a concern for aspiration postoperatively. In each scenario, an intensive care unit bed was not obtained until the day of the procedure, either just before or during the actual surgery. When adjusted for age, sex, body mass index, ASA physical status, presenting symptoms, concomitant syringomyelia, previous decompression surgery and year of surgery the odds ratio for an unanticipated admission to the intensive care unit following an intraoperative anesthetic‐related complication was 5.9 (95% CI 0.51–59.6, *p* = .149) (Table 3). The number of patients needed to harm was 156 and when corrected for confounders was calculated to be 212.

**TABLE 3:** Firth's bias reduction logistic regression model for an unanticipated intensive care unit admission

Variable	*β*	Odds ratio	95% Confidence intervals	*p*
Age (years)	−.10	0.89	0.69–1.13	.357
Male sex	−.48	0.62	0.04–4.42	.641
Body mass index	.04	1.04	0.96–1.12	.189
ASA physical status >2	2.15	8.60	0.99–1010	.050
Presenting symptom				
Incidental		1	‐‐	
Neurological	−1.68	0.18	0.00–4.72	.291
Clinical comorbidity	−.31	0.73	0.06–12.50	.806
Concomitant syringomyelia	−.56	0.58	0.04–5.85	.626
Intraoperative complication	1.77	5.87	0.51–59.63	.149
Previous decompression surgery	.11	1.11	0.06–10.91	.930
Year of Surgery				
2009–2015		1		
2016–2021	−1.56	0.21	0.00–1.95	.190
Intercept	−3.34			.020

There were thirteen planned intensive care unit admissions for various reasons, mostly for airway protection and concerns with ventilation. Two patients had a tracheostomy requiring mechanical ventilation around the clock, two patients required bilevel positive airway pressure (BiPap) (one with a history of asthma, vocal cord paresis, and aspiration pneumonia and the other with recently diagnosed central sleep apnea), and two patients required CPAP postoperatively (one for obstructive sleep apnea [OSA] and one with OSA and Goldenhar syndrome). One patient had a diagnosis of combined central and obstructive sleep apnea that did not require any noninvasive forms of mechanical ventilation and had a decreased gag reflex. Another patient had a history of OSA and stridor while sleeping, gastroesophageal reflux disease, and vocal cord dysfunction. One patient had a history of obesity and difficulty swallowing while another patient had a history of difficulty with clearing of secretions. One patient had an active parainfluenza infection with a history of asthma and a recent RSV bronchiolitis. One patient had a history of heart surgery for transposition of the great arteries, ventricular septal defect and atrial septal defect, as well as seizures, asthma, a recent pneumonia, and sinus bradycardia. Finally, one patient had a history of moderate idiopathic pulmonary hypertension. All these patients had anticipated increased postoperative monitoring needs that led to a planned intensive care unit admission.

Postoperative complications in our patient cohort are listed in Table 4. The most frequent postoperative complications included nausea and/or vomiting (38 of 296, 12.8%), uncontrolled pain (6 of 296, 2.0%), and apnea requiring oxygen therapy (6 of 296, 2.0%). Upper airway obstruction was more common in patients with unanticipated admission to the intensive care unit, but only present in 3 of 296 (1.0%) patients. Surgical outcomes included neurological improvement in 264 patients (89.2%), and complete resolution of a syrinx in 30 of 169 children presenting with this finding (17.7%). Other surgical outcomes included aseptic meningitis, cerebrospinal fluid leak, new‐onset hydrocephalus, pseudomeningocele, wound infection, epidural or subdural hematoma. There were no differences in surgical outcomes in patients with and without an unanticipated admission to the intensive care unit. Emergency room visits within 7 days of surgery and readmission within 30 days of discharge were not different in patients with an unanticipated admission to the intensive care unit versus those that did not.

**TABLE 4:** Postoperative complications and surgical outcomes

	Unanticipated intensive care unit admission		Difference (95% Confidence interval)	*p*
	No	Yes		
	*n* = 291	*n* = 5		
Postoperative complications				
Nausea/vomiting	37 (13)	1 (20)	8 (−49 to 35)	.499
Uncontrolled pain	6 (2)	0 (0)	2 (−6 to 27)	.746
Apnea requiring oxygen therapy	5 (2)	1 (20)	−18 (−6 to 27)	.167
Blood transfusion for anemia	4 (1)	0 (0)	1 (−43 to 26)	.724
Infection	4 (1)	0 (0)	1 (−43 to 26)	.792
Upper airway obstruction	1 (1)	2 (40)	−39 (−93 to −26)	.002
Pneumonia	1 (1)	0 (0)	1 (−45 to 26)	.861
Surgical Outcomes				
Neurological improvement				
Yes	260 (90)	4 (80)	10 (−35 to 54)	
No	19 (6)	1 (20)	−14 (−59 to 32)	.670
Lost to follow‐up	3 (1)	0 (0)	1 (−44 to 26)	
No preoperative deficit	8 (3)	0 (0)	3 (−40 to 26)	
New or worsening neurological deficit				
No	273 (94)	5 (100)	−6 (−15 to 3)	
Yes	16 (5)	0 (0)	5 (−35 to 26)	.850
Recurrence of old symptoms	2 (1)	0 (0)	1 (−44 to 26)	
Complete syrinx resolution *n* (%)				
Yes	29 (17)	1 (100)	−83 (−100 to 59)	
No	111 (66)	0 (0)	66 (18–93)	.206
Lost to follow‐up	28 (27)	0 (0)	27 (−26 to 39)	
Pseudomeningocele	22 (8)	1 (20)	−12 (−57 to 33)	.335
Aseptic meningitis	6 (2)	0 (0)	2 (−42 to 26)	.760
Cerebrospinal fluid leak	8 (3)	0 (0)	3 (−40 to 26)	.637
New‐onset hydrocephalus *n* (%)	8 (3)	0 (0)	3 (−40 to 26)	.707
Epidural or subdural hematoma	3 (1)	0 (0)	1 (−44 to 26)	.819
Wound infection	5 (1)	0 (0)	1 (−10 to 13)	.726
Emergency room visit within 7 days of surgery	27 (9)	2 (40)	−31 (−83 to 22)	.077
Readmission within 30 days	33 (11)	1 (20)	−9 (−52 to 35)	.459

*Note*: Data reported as *n* (% of column). Differences reported as percent difference and 95% confidence intervals.

## 4 DISCUSSION

The results of our study demonstrate a low incidence of intraoperative anesthesia‐related complications in children undergoing posterior fossa decompression for Chiari I Malformation. Nevertheless, we found an association between ASA physical status >2, younger age, and an anesthesia‐related intraoperative complication with unanticipated intensive care unit admission. An intraoperative anesthesia‐related complication was associated with an increased odds ratio and a number needed to harm of 156 for an unanticipated admission to the intensive care unit admission. A duraplasty was not associated with an increased odds ratio for an unanticipated admission to the intensive care unit and did not reflect an incidence of intraoperative anesthesia‐related complications.

Very few studies have investigated anesthesia‐related complications of posterior fossa decompression in children. Jones et. al reported bradycardia in three of 24 patients during plugging of the obex, a neurosurgical maneuver for Chiari management that is no longer routinely performed.^11^ Of 22 investigations examining the clinical outcomes of surgery for Chiari I Malformation (Appendix S2), only two studies reported complications which may be related to anesthesia. One of 24 patients had postoperative nausea in one study,^12^ and another trial noted ventilatory failure requiring reintubation in two of 43 patients.^13^ Our study, which specifically looked at complications related to anesthesia for this procedure, confirmed the minimal anesthetic complications noted by prior investigations. Major surgical complications following posterior fossa decompression surgery include aseptic meningitis,^14^, ^15^, ^16^, ^17^, ^18^, ^19^ cerebrospinal fluid leak,^11^, ^12^, ^14^, ^17^, ^18^, ^19^, ^20^, ^21^, ^22^, ^23^, ^24^, ^25^ pseudomeningocele,^11^, ^12^, ^14^, ^16^ and wound infection.^12^, ^16^, ^17^, ^20^ We found similar incidences of these complications in our cohort (see Table 4), with satisfactory long‐term surgical outcomes and overall good prognosis in our patient population.

While only five patients in our cohort had unanticipated intensive care unit admissions, two of them had intraoperative anesthesia‐related complications while three did not. All three without an intraoperative anesthesia‐related complication could have been planned intensive care unit admissions with improved preoperative recognition and planning, as many patients with similar medical problems had intensive care unit beds reserved for them ahead of time. Of the two patients admitted to intensive care with intraoperative complications, both had chronic medical conditions; however, they likely may not have needed intensive care admission had those intraoperative anesthesia‐related complications not occurred that triggered the intensive care admission. There are some criteria at our institution necessitating the need for postoperative intensive care, including the need for postoperative mechanical ventilation, whether invasive or noninvasive. This was the reason for several of the planned intensive care unit admissions, while the rest were mostly based off clinical decision‐making, either by the neurosurgeon, the anesthesiologist, or both. Our intensive care unit is willing to accept almost any postoperative admission that is deemed necessary by the attendings surgeon or anesthesiologist, if there is a clinically relevant reason, which were present in these cases.

The results of our study should be interpreted in the context of its limitations. This study was retrospective in nature, with inherent deficits related to this methodology. Our dataset is also limited to patients treated between 2009 and 2021 at a single tertiary care medical center. While it is possible that current surgical and anesthetic management techniques may have changed since this time period, we did not find any differences in the incidence or trend in the rate of unanticipated intensive care unit admission or intraoperative anesthesia‐related complication throughout the years of this study. Interestingly, there were no unanticipated intensive care unit admissions from 2017 to 2021. While not specifically evaluated, we speculate this change occurred due to the increased awareness of elevation of postoperative care to the intensive care unit as a quality metric during that time period. Some of these intraoperative complications may have been directly caused by surgical factors (compression of brainstem, significant bleeding) or anesthetic factors (inappropriate medication administration, anesthetic too deep); however, we are unable to determine a cause‐and‐effect relationship between an event and a complication.

In summary, we found relative low rates of an unanticipated intensive care admission or an intraoperative anesthesia‐related complication in children undergoing posterior fossa decompression for Chiari I Malformation. Younger patients and those with ASA physical status >2 are associated with an increase in an unanticipated intensive care unit admission. Anesthesia‐related complications were also associated with an increased odds for an unanticipated intensive care admission that was reduced when adjusted for confounding factors, but still represented a number needed to harm of 212. Particular consideration could be made for younger patients or those with medical conditions with systemic involvement. This information could help guide future clinical decisions in the perioperative care of this surgical population, especially when considering appropriate intraoperative invasive monitoring and intravenous access during the surgical procedure, as well as appropriate planning for postoperative disposition and care.

## CONFLICT OF INTEREST

The authors have no conflicts of interest to declare.

## ETHICAL APPROVAL

This study was approved by the Institutional Review Board of the Ann & Robert H. Lurie Children's Hospital of Chicago, IRB number: IRB 2016–110, approval date 17 June 2018, principal investigator Hubert A. Benzon.

## Supporting information


Appendix S1
Appendix S2

## Data Availability

The data that support the findings of this study are available from the corresponding author upon reasonable request.
