# Supplementary material for: Patient and operative factors associated with unanticipated intensive care admission and outcomes following posterior fossa decompressions in children: A retrospective study
Source: Paediatr Anaesth. 2022 Jun 3;32(8):937–45. doi: 10.1111/pan.14496 (PMC9541405; doi:10.1111/pan.14496)
Supplement: Supplementary file 1 — Appendix S1 Appendix S2 [file PAN-32-937-s001.docx]

Appendix S1

Table S1: Presenting symptom or reason for evaluation of Chiari I Malformation (n=296).

| Neurologic  Headache  Weakness/numbness  Seizure  Vision changes  Emesis  Gait disturbances  Brainstem/ Intracranial pressure symptoms  Behavioral changes  Clinical co-morbidity  Scoliosis  Back/neck pain  Comorbidity  Existing Chiari evaluation  Existing cervical stenosis evaluation  Incidental finding | 182 (62)  132 (72)  17 (9)  11 (6)  10 (5.5)  4 (2.5)  4 (2.5)  3 (2)  1 (0.5)  92 (31)  43 (47)  16 (17)  16 (17)  15 (17)  2 (2)  22 (7) |
| --- | --- |

Data reported as n (% of column)

**Appendix S2. References of investigations examining clinical outcomes of surgery for Chiari I Malformation.**

1. Hoffman HJ, Neill J, Crone KR, Hendrick EB, Humphreys RP: Hydrosyringomyelia and its management in childhood. Neurosurgery 1987;21:347–351.

2. Nagib MG: An approach to symptomatic children (ages 4-14 years) with Chiari type I malformation. Pediatr Neurosurg 1994;21:31–35.

3. Weinberg JS, Freed DL, Sadock J, Handler M, Wisoff JH, Epstein FJ: Headache and Chiari I malformation in the pediatric population. Pediatr Neurosurg 1998;29:14–18.

4. Krieger MD, McComb JG, Levy ML: Toward a simpler surgical management of Chiari I malformation in a pediatric population. Pediatr Neurosurg 1999;30:113–121.

5. James HE, Brant A: Treatment of the Chiari malformation with bone decompression without durotomy in children and young adults. Childs Nerv Syst 2002;18:202–206.

6. Lazareff 2002 Lazareff JA, Galarza M, Gravori T, Spinks TJ: Tonsillectomy without craniectomy for the management of infantile Chiari I malformation. J Neurosurg 2002;97:1018–1022.

7. Greenlee JD, Donovan KA, Hasan DM, Menezes AH: Chiari I malformation in the very young child: the spectrum of presentations and experience in 31 children under age 6 years.

Pediatrics 2002;110:1212–1219.

8. Limonadi FM, Selden NR: Dura-splitting decompression of the craniocervical junction: reduced operative time, hospital stay, and cost with equivalent early outcome. J Neurosurg

2004;101 (2 Suppl):184–188.

9. Navarro R, Olavarria G, Seshadri R, Gonzales-Portillo G, McLone DG, Tomita T: Surgical results of posterior fossa decompression for patients with Chiari I malformation. Childs Nerv Syst 2004;20:349–356.

10. Yeh DD, Koch B, Crone KR: Intraoperative ultrasonography used to determine the extent of surgery necessary during posterior fossa decompression in children with Chiari I malformation Type I. J Neurosurg 2006;105 (1 Suppl):26–32.

11. Caldarelli M, Novegno F, Vassimi L, Romani R, Tamburrini G, Di Rocco C: The role of limited posterior fossa craniectomy in the surgical treatment of Chiari malformation Type I: experience with a pediatric series. J Neurosurg 2007;106 (3 Suppl):187–195.

12. McGirt MJ, Attenello FJ, Datoo G, Gathinji M, Atiba A, Weingart JD, et al: Intraoperative ultrasonography as a guide to patient selection for duraplasty after suboccipital decompression in children with Chiari malformation Type I. J Neurosurg Pediatr 2008;2:52–57.

13. Attenello FJ, McGirt MJ, Garcés-Ambrossi GL, Chaichana KL, Carson B, Jallo GI: Suboccipital decompression for Chiari I malformation: outcome comparison of duraplasty with expanded polytetrafluoroethylene dural substitute versus pericranial autograft. Childs Nerv Syst 2009;25:183–190.

14. Albert GW, Menezes AH, Hansen DR, Greenlee JD, Weinstein SL: Chiari malformation Type I in children younger than age 6 years: presentation and surgical outcome. J Neurosurg Pediatr 2010;5:554–561.

15. Mottolese C, Szathmari A, Simon E, Rousselle C, Ricci-Franchi AC, Hermier M: Treatment of Chiari type I malformation in children: the experience of Lyon. 2011;Neurol Sci 32 (Suppl 3):S325–S330.

16. Parker SR, Harris P, Cummings TJ, George T, Fuchs H, Grant G: Complications following decompression of Chiari malformation Type I in children: dural graft or sealant? J Neurosurg Pediatr 2011;8:177–183.

17. Valentini L, Visintini S, Saletti V, Chiapparini L, Estienne M, Solero CL: Treatment for Chiari 1 malformation (CIM): analysis of a pediatric surgical series. Neurol Sci 2011;32 (Suppl 3):S321–S324.

18. Tubbs RS, Beckman J, Naftel RP, Chern JJ, Wellons JC III, Rozzelle CJ, et al: Institutional experience with 500 cases of surgically treated pediatric Chiari malformation Type I. J Neurosurg Pediatr 2011;7:248–256.

19. Bollo RJ, Riva-Cambrin J, Brockmeyer MM, Brockmeyer DL: Complex Chiari malformations in children: an analysis of preoperative risk factors for occipitocervical fusion. J Neurosurg Pediatr 2012;10:134–141.

20. Chen JA, Coutin-Churchman PE, Nuwer MR, Lazareff JA: Suboccipital craniotomy for Chiari I results in evoked potential conduction changes. Surg Neurol Int 2012;3:165.

21. Litvack ZN, Lindsay RA, Selden NR: Dura splitting decompression for Chiari I malformation in pediatric patients: clinical outcomes, healthcare costs, and resource utilization. Neurosurgery 2013;72:922–929.

22. Scott WW, Fearon JA, Swift DM, Sacco DJ: Suboccipital decompression during posterior cranial vault remodeling for selected cases of Chiari malformations associated with craniosynostosis. J Neurosurg Pediatr 2013;12:166–170.
